# Integrated Analyses of Metabolome and RNA-seq Data Revealing Flower Color Variation in Ornamental *Rhododendron simsii* Planchon

**DOI:** 10.3390/genes15081041

**Published:** 2024-08-07

**Authors:** Zhiliang Li, Siduo Xu, Hongmei Wu, Xuchun Wan, Hanhan Lei, Jiaojun Yu, Jun Fu, Jialiang Zhang, Shuzhen Wang

**Affiliations:** 1 College of Biology and Agricultural Resources, Huanggang Normal University, Huanggang 438000, China; lizhiliang04@163.com (Z.L.);; 2 Department of Library, Huanggang Normal University, Huanggang 438000, China; wuhongmei2015@163.com

**Keywords:** *Rhododendron simsii* Planchon, anthocyanins, flower color variation, molecular mechanism, flower color breeding

## Abstract

*Rhododendron simsii* Planchon is an important ornamental species in the northern hemisphere. Flower color is an important objective of *Rhododendron* breeding programs. However, information on anthocyanin synthesis in *R. simsii* is limited. In this research, the regulatory mechanism of anthocyanin biosynthesis in *R. simsii* was performed through the integrated analysis of metabolome and RNA-seq. A total of 805 and 513 metabolites were screened by positive and negative ionization modes, respectively, In total, 79 flavonoids contained seven anthocyanidins, 42 flavanones, 10 flavans, 13 flavones, and seven flavonols. Methylated and glycosylated derivatives took up the most. Differentially accumulated metabolites were mainly involved in “flavone and flavonol biosynthesis”, “cyanoamino acid metabolism”, “pyrimidine metabolism”, and “phenylalanine metabolism” pathways. For flavonoid biosynthesis, different expression of *shikimate O-hydroxycinnamoyltransferase*, *caffeoyl-CoA O-methyltransferase*, *flavonoid 3′-monooxygenase*, *flavonol synthase*, *dihydroflavonol 4-reductase/flavanone 4-reductase*, *F3′5′H*, *chalcone synthase*, *leucoanthocyanidin reductase*, and *5-O-(4-coumaroyl)-D-quinate 3′-monooxygenase* genes ultimately led to different accumulations of quercetin, myricetin, cyanidin, and eriodictyol. In flavone and flavonol biosynthesis pathway, differential expression of *F3′5′H*, *flavonoid 3′-monooxygenase* and *flavonol-3-O-glucoside/galactoside glucosyltransferase* genes led to the differential accumulation of quercetin, isovitexin, and laricitrin. This research will provide a biochemical basis for further modification of flower color and genetic breeding in *R. simsii* and related *Rhododendron* species.

## 1. Introduction

*R. simsii* (2n = 26), a member of the vascular *Rhododendron* genus, is mainly distributed in the northern hemisphere, especially in Asia and Europe [1]. As an outstanding ornamental species, the perennial shrub *R. simsii* is vital for ecotourism and forest wellness [2,3]. *R. simsii* is also used for natural medicines due to significant biological activities of diverse metabolites, especially in treating gynecological diseases. Flower bud variation is a well-known phenomenon widely existing in Belgian hybrids of *R. simsii* [4]. Wild *R. simsii* germplasm resources are often used as breeding parents for cultivating breakthrough varieties due to their beautiful tree shape and rich flower colors. In particular, different accumulations of flavonoids in petals lead to the rich flower colors in wild *R. simsii* germplasm resources. It is worth noting that flower color is largely determined by metabolic composition, especially flavonoids, carotenoids, and betalains [5].

Flavonoids, providing flower colors ranging from pale yellow to blue, could be classified into six structural types, containing flavonols, flavanones, chalcones, flavones, flavan-3-ols, and anthocyanins [6,7]. Anthocyanins, water-soluble pigments derived from a branched flavonoid biosynthetic pathway, could confer on plants blue, purple, and red colors [8,9,10]. Moreover, anthocyanins were synthesized in an endoplasmic reticulum and then sequestered into a vacuole [11,12]. In particular, anthocyanins are beneficial in protecting plants against stress conditions, oxidant damage, and infection by pathogens [13,14]. Anthocyanin derivatives are common in vascular plants containing pelargonidin (Pg), cyanidin (Cy), peonidin (Pn), malvidin (Mv), petunidin (Pt), and delphinidin (Dp) [15]. In regard to the flavonoid biosynthesis pathway, anthocyanins and proanthocyanins (PAs) are two end products [16,17]. In particular, anthocyanin accumulation is a complex process involving a series of enzymes, such as chalcone synthase (CHS), flavanone 3-hydroxylase (F3H), phenylalanine ammonia-lyase (PAL), chalcone isomerase (CHI), flavonoid 3′-hydroxylase (F3′H), flavonoid 3′,5′-hydroxylase (F3′5′H), anthocyanidin synthase (ANS), dihydroflavonol 4-reductase (DFR), and anthocyanidin reductase (ANR) [18].

Dynamic alteration in the expression pattern of anthocyanin biosynthesis genes will greatly affect the accumulation of anthocyanins [19]. Quercetin, myricetin, mearnsetin, kaempferol, isorhamnetin, as well as diosmetin and their glycoside derivatives have been isolated and elucidated in some *Rhododendron* species [20,21]. In particular, cyanidin 3,5-dimonoside, cyanidin 3-glycoside, cyanidin 3,5-diglycoside, cyanidin 3-monoside, and quercetin have been isolated from *Rhododendron* varieties [4]. Quercetin 3-glucoside and quercetin 3-rhamnoside have been isolated from the reddish-purple blotch areas of *R. simsii* flowers [22]. Based on LC-ESI-MS/MS and a broad-targeted metabolomic approach, a total of 149 flavonoids and their glycosylated or methylated derivatives were identified in *Rhododendron pulchrum* Sweet, containing 18 anthocyanins and 32 flavonols [23]. New pigment synthesis caused by a change in either anthocyanins or flavonols often leads to new varieties. However, the systematic analysis of secondary metabolites of the *Rhododendron* species is still very limited, hindering corresponding genetic improvement of flower color.

Metabolomics, analyzing metabolites comprehensively, shows great potential in elucidating metabolic processes. To deeply understand the pigment accumulation patterns in *R. simsii* flowers, metabolite interactions and phenotypic variation among three *R. simsii* varieties with different flower colors were analyzed. Moreover, integrated analysis of metabolomics and mRNA-seq data were also performed, aiming to clarify features of pigment composition and provide a basis for genetic improvement in *R. simsii* and related species.

## 2. Materials and Methods

### 2.1. Plant Materials

**Figure 1:**
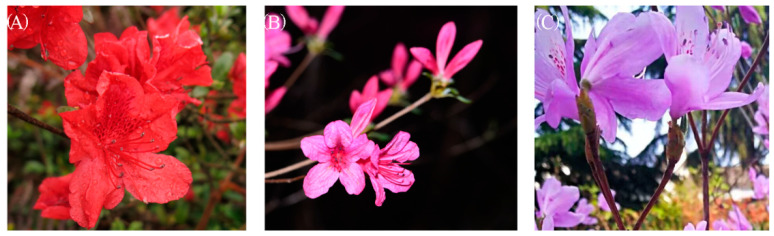
Flower tissues of *R. simsii* varieties: (**A**) “red variety”; (**B**) “pink variety”; (**C**) “violet variety”.

Wild and healthy *R. simsii* plants (“red variety”, “pink variety”, and “violet variety”) were selected from the shrub ecosystem of the Dabie Mountains (30°43′19″–31°04′28″ N, 116°23′11″–116°21′24″ E, 956–1015 m). These specimens were identified by Professor Yuanping Fang (a botanist from Huanggang Normal University). According to the Royal Horticultural Society color chart, flower colors of different *R. simsii* varieties were evaluated. The red, pink, and violet flowers of *R. simsii* were separately sampled from different individual plants (Figure 1). Each sample has three biological repeats. These samples were frozen in liquid nitrogen immediately and stored at −80 °C until further usage. All materials were well conserved at the Huanggang Normal University Herbarium (Huanggang, Hubei Province, China).

### 2.2. Sample Preparation and LC-MS/MS Analysis

The freeze-dried petals were ground, and 100 mg powder was extracted overnight at 4 °C with 0.5 mL of 80% aqueous methanol to ensure the extraction efficiency of the metabolites. Following centrifugation (15,000× *g* 20 min), the extracts were filtered and then analyzed with an LC-ESI-QTRAP-MS/MS system. Chromatographic analysis was performed with a HypesilGoldcolumn (C18) at the column temperature of 40 °C. The flow rate was set as 0.2 mL/min. The solvent system under positive pressure consisted of solvent A (water with 0.1% formic acid) and solvent B (methanol). Moreover, the solvent system under negative pressure was set as follows: solvent A (water with 5mM ammonium acetate, pH 9.0) and solvent B (methanol). Three quality control (QC) samples were prepared through mixing 9 experimental samples with an equal volume, which were tested before, during, and after the injection of the experimental samples. The gradient program was set as follows: 1.5 min, 98% solvent A and 2% solvent B; 13 min, 100% solvent B; 33.10 min, 98% solvent A and 2% solvent B.

Effluent was connected to the ESI-HRMS, and ESI source operation parameters were set as follows: spray voltage (IS) 3.5 kV; sheath gas flow rate, 35 psi; aux gas flow rate, 10 L/min; capillary temperature, 320 °C; S-lens RF level, 60; aus gas heater temperature, 350 °C; polarity: positive and negative; data-dependent scans. The whole procedure was repeated three times. In particular, record data were controlled with MassHunter Qualitative Analysis B.07 software (Agilent Technologies, Palo Alto, CA, USA). Analyst 1.6.2 software (AB Sciex, Darmstadt, Germany) was used for data acquisition, peak integration, and calculations. The quantification of metabolites was carried out in a multiple reaction monitoring model. In particular, metabolites detected under positive and negative ionization modes were renamed as “pos metabolites” and “neg metabolites”, respectively. 

### 2.3. Identification of Metabolites

The R package MetaX was used to transform the metabolomic data, which were then subjected to principal component analysis (PCA) and partial least squares-discriminate analysis (PLS-DA). In particular, the metabolite data were log2 transformed to improve normality and then subjected for hierarchical clustering analysis (HCA). The obtained VIP values (variable importance for the projection > 1) and FC scores (fold-change ≥ 2 or ≤0.5) were used to screen differentially accumulated metabolites. Compounds were identified through comparing *m*/*z* values, retention time (RT), and fragmentation patterns with the mzCloud (https://www.mzcloud.org/, accessed on 9 September 2023), mzVault, and Masslist databases. For metabolites without standards, fragmentation patterns similar to identified metabolites were queried MS2 spectral data or searched against the MassBank database [23]. The Kyoto Encyclopedia of Genes and Genomes (KEGG, https://www/genome.jp/kegg/pathway.html, accessed on 12 September 2023), HMDB (https://hmdb.ca/metabolites, accessed on 15 September 2023), and LIPID MAPS (http://www.lipidmaps.org/, accessed on 9 October 2023) were used for annotation of the metabolites.

### 2.4. Integration Analysis of Metabolomics and RNA-seq Data 

RNA-seq data of the same *R. simsii* varieties under the BioProject, Biosample, and SRA numbers of PRJNA998472, SAMN36704916-SAMN36704933, and SRR25433158-SRR25433172 were used for integration analysis. According to FPKM (Fragments per kb per million mapped reads) method, the expression levels of assembled unigenes were accounted for. Differentially expressed genes were screened out with the DESeq2 R package (Bioconductor 3.19) [24]. Differentially expressed genes and differentially accumulated metabolites were mapped to the KEGG pathway simultaneously. The *p*-value was calculated through a hyper-geometric test. Functional and pathway enrichment analyses were also carried out, aiming at evaluating the significance of GO terms and KEGG pathways. Heatmaps were constructed with ComplexHeatmap (version 2.10.0). 

## 3. Results

### 3.1. Identification of Metabolites in Three Varieties of R. simsii

Under the parameters of VIP > 1.0, FC > 1.2 or FC < 0.833, and *p*-value < 0.05, 805 and 513 metabolites were screened out under the positive and negative ion modes, respectively (Table S1). 

#### 3.1.1. Annotation with HMDB Database

**Figure 2:**
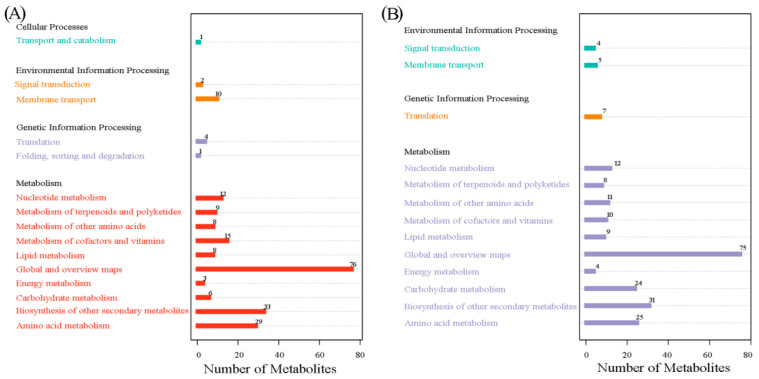
Annotation of metabolites detected under positive ionization mode (**A**) and negative ionization mode (**B**) against HMDB database.

The HMDB database was used to analyze molecular metabolite annotation and enzyme or transporter associated and related properties. In total, 394 “pos metabolites” were divided into 10 classes: “alkaloids and derivatives” (6); “benzenoids” (45); “lignans, neolignans, and related compounds” (7); “lipids and lipid-like molecules” (95); “nucleosides, nucleotides, and analogues” (13); “organic acids and derivatives” (55); “organic nitrogen compounds” (9); “organic oxygen compounds” (34); “organoheterocyclic compounds” (63), and “phenylpropanoids and polyketides” (67) (Table S2). For KEGG pathway analysis, 217 metabolites could be mapped, containing “cellular processes” (one pathway), “environmental information processing” (two pathways), “genetic information processing” (two pathways), and “metabolism” (ten pathways). In particular, “global and overview maps” and “biosynthesis of other secondary metabolites” were the most enriched KEGG pathways (Figure 2A).

Furthermore, a total of 287 “neg metabolites” could be annotated by the HMDB database (Table S2): “benzenoids” (30); “lignans, neolignans, and related compounds” (4); “lipids and lipid-like molecules” (79); “nucleosides, nucleotides, and analogues” (13); “organic acids and derivatives” (34); “organic oxygen compounds” (38); “organoheterocyclic compounds” (20); and “phenylpropanoids and polyketides” (69). In particular, 225 metabolites could be mapped to the corresponding 13 KEGG pathways, including “environmental information processing” (two pathways), “genetic information processing” (one pathway), and “metabolism” (ten pathways). Similarly, “global and overview maps” and “biosynthesis of other secondary metabolites” were the most enriched KEGG pathways (Figure 2B). 

#### 3.1.2. Lipid Map 

**Figure 3:**
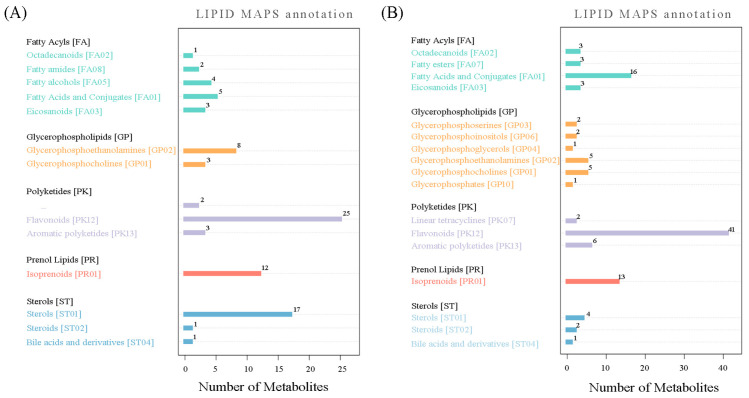
LIPID MAPS annotation of metabolites detected under positive ionization mode (**A**) and negative ionization mode (**B**).

For the LIPID MAPS annotation, 197 metabolites could be mapped, including 110 “pos metabolites” and 87 “neg metabolites” (Figure 3 and Table S3). In particular, these 197 metabolites could be divided into five categories: “fatty acyls”, “glycerophospholipids”, “polyketides”, “prenol lipids”, and “sterols”. For the “fatty acyls” category, “fatty acids and conjugates” was the main class: “fatty esters” was only found in “neg metabolites”, while “fatty amides” and “fatty alcohols” existed only for “pos metabolites”. For the “glycerophospholipids” category, five classes could be identified related to “neg metabolites”, while only two classes were classified for “pos metabolites”. Along with “flavonoids” and “aromatic polyketides”, the “linear tetracyclines” class was also mapped for the “polyketides” category in regard to “neg metabolites”. For the “prenol lipids” category, only “isoprenoids” was found. Regarding the “sterols” category, three classes were mapped, including “sterols”, “steroids”, and “bile acids and derivatives”. 

#### 3.1.3. Pigment Identified in Three *R. simsii* Varieties

**Figure 4:**
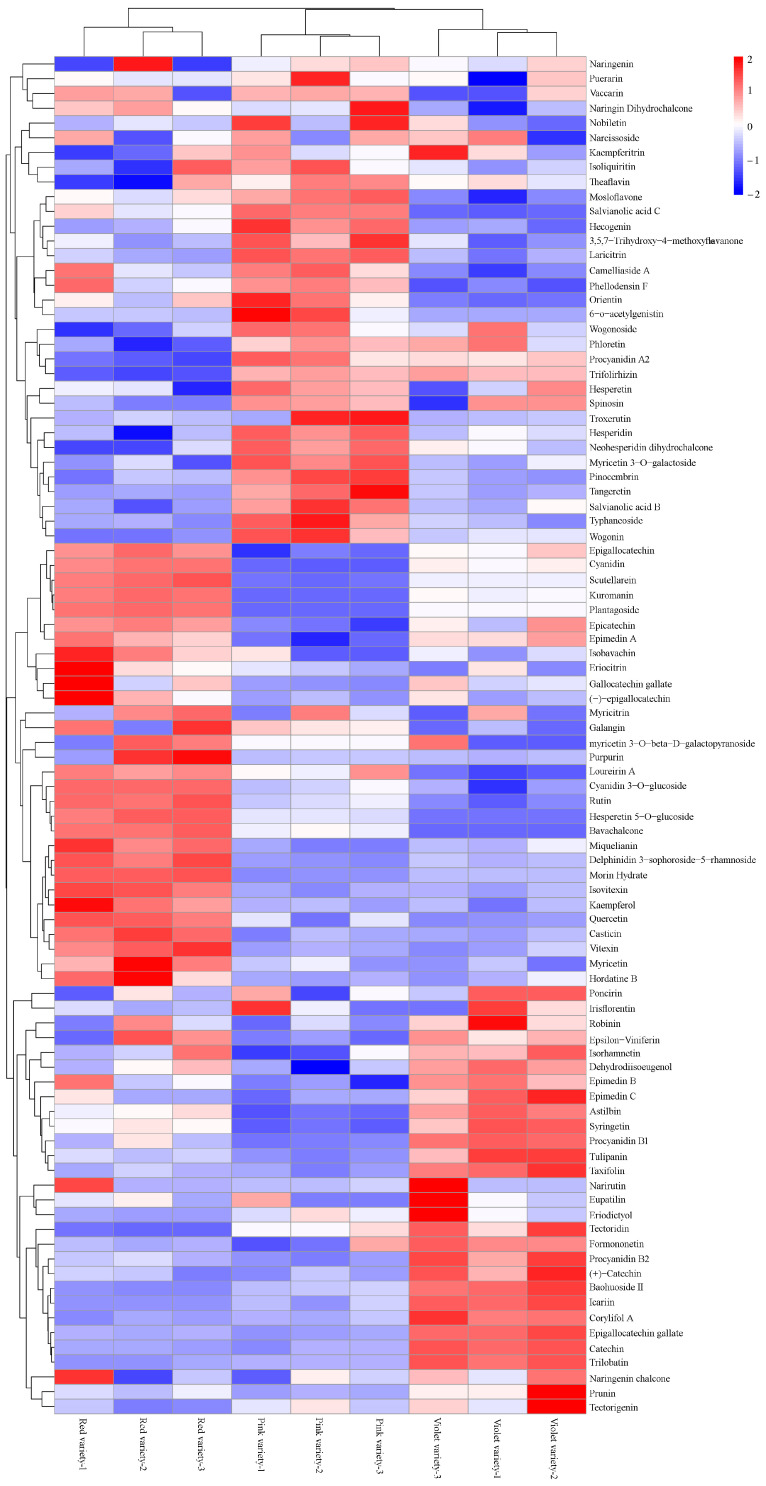
Cluster analyses of variety-specific accumulation patterns.

In total, 79 flavonoids were obtained, containing seven anthocyanidins, 42 flavanones, 10 flavans, 13 flavones, and seven flavonols. Moreover, isoflavonoids (eight) and 2-arylbenzofuran flavonoids (five) were also identified. For the six common types of anthocyanin derivatives, only Cy and Dp were screened in three *R. simsii* varieties (Table 1). The other four types, including Pg, Pn, Mv, and Pt, were not found. Furthermore, kuromanin, an anthocyanidin-3-O-glycoside metabolite, was also the main anthocyanin for *R. simsii* flowers. Along with three procyanidin (A2, B1, and B2), tulipanin was also identified as a proanthocyanidin (Table S4). In regard to flavanones, methylated and glycosylated derivatives took up the most. Considering flavans, catechins and epigallocatechins were the most representative. Based on the variety-specific accumulation patterns, flavonoids and derivatives could be clearly grouped into three main clusters with six sub-clusters (Figure 4). 

### 3.2. Screening of Differential Metabolites among Different *R. simsii* Varieties 

**Figure 5:**
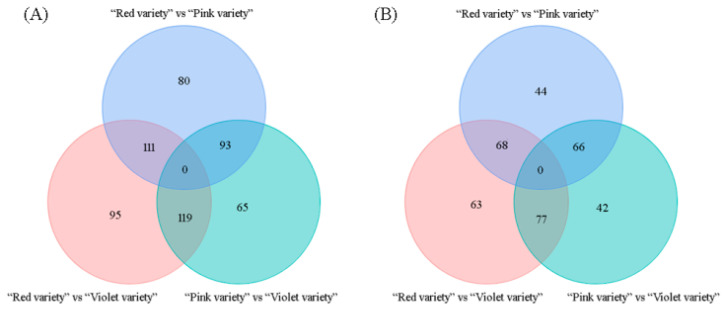
Venn diagram of differentially accumulated metabolites detected under positive ionization mode (**A**) and negative ionization mode (**B**).

**Figure 6:**
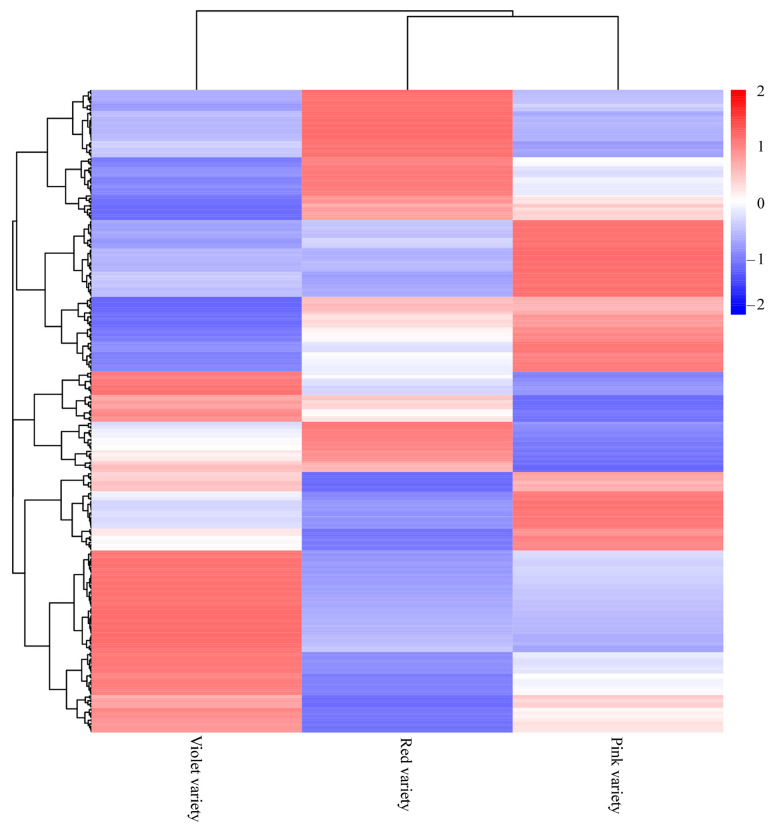
Cluster analyses of three *R. simsii* varieties based on metabolite profile.

In order to identify critical metabolites responsible for variation in flower color, pairwise comparisons and PLS-DA were performed. Based on FC scores and VIP values, a list of 923 differentially accumulated metabolites were obtained (Table S5). For these differentially accumulated “pos metabolites”, 80, 95, and 65 metabolites were only accumulated in the “red variety”, “pink variety”, and “violet variety”, respectively (Figure 5A). In regard to differentially accumulated “neg metabolites”, 44, 63, and 42 metabolites were merely present in the “red variety”, “pink variety”, and “violet variety”, respectively (Figure 5B). Signal intensity of these detected metabolites was relative to the corresponding abundance. The accumulation of metabolites displayed obvious, clear phenotypic variation. Based on metabolic profile, the “red variety” and “pink variety” were clustered together, while the “violet variety” was clustered alone (Figure 6). According to PCA results, flower variation was metabolite-related (Figure S1).

#### 3.2.1. Differential Metabolites between “Red Variety” and “Pink Variety” 

**Figure 7:**
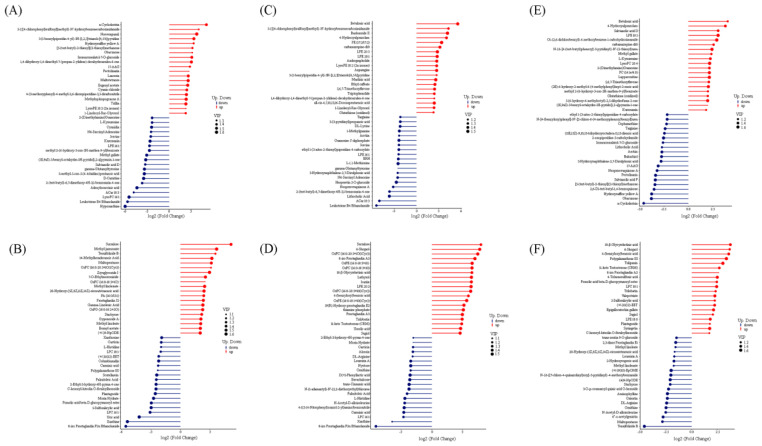
Typical representatives of three *R. simsii* varieties: (**A**) “red variety” vs. “pink variety” under positive ionization mode; (**B**) “red variety” vs. “pink variety” under negative positive ionization mode; (**C**) “red variety” vs. “violet variety” under positive ionization mode; (**D**) “red variety” vs. “violet variety” under negative ionization mode; (**E**) “pink variety” vs. “violet variety” under positive ionization mode; (**F**) “pink variety” vs. “violet variety” under negative ionization mode.

Upon comparison of the “red variety” and “pink variety”, a total of 462 differentially accumulated metabolites were enriched, and the accumulation of 293 metabolites was upregulated, while that of 169 metabolites was downregulated (Table S6). For these 284 differentially accumulated “pos metabolites”, 193 metabolites were upregulated with the typical representatives of α-Cyclodextrin, norverapamil, 3-(1-benzylpiperidin-4-yl)-3H-[1,2,3]triazolo [4,5-b] pyridine, hydroxysafflor yellow A, 3-{[(4-chlorophenyl)sulfonyl]methyl}-N”-hydroxybenzenecarboximidamide, and [5-(tert-butyl)-2-thienyl] (2-thienyl)methanone; 91 metabolites were downregulated with representatives of hypoxanthine, leukotriene B4 Ethanolamide, lysoPC 16:1, ACar 18:3, and adenylosuccinic acid (Figure 7A and Table S6). In regard to 178 differentially accumulated “neg metabolites”, 100 metabolites were upregulated, mainly containing sucralose, methyl jasmonate, tenuifoliside B, 14-Methylhexadecanoic acid, and maltopentaose; 78 metabolites were downregulated with representatives of 8-iso prostaglandin F2α ethanolamide, xanthine, uric acid, LPC 16:1, and 5-sulfosalicylic acid (Figure 7B and Table S6). 

#### 3.2.2. Differential Metabolites between “Red Variety” and “Violet Variety”

Upon comparison of the “red variety” and “violet variety”, 533 differentially accumulated metabolites were identified, among which 311 metabolites were upregulated, while 222 metabolites were downregulated (Table S7). In particular, 325 ”pos metabolites” and 208 neg metabolites“ were found to be differentially accumulated. For differentially accumulated metabolites detected under the positive mode, the main upregulated metabolites contained betulonic acid, 3-{[(4-chlorophenyl)sulfonyl]methyl}-N’-hydroxybenzene-carboximidamide, PE (17:2/17:2), baohuoside II, LPE 20:3, carbamazepine-d10, and 4-Hydroxyalprazolam, while the typical downregulated metabolites were main leukotriene B4 Ethanolamide, ACar 18:3, lithocholic acid, 2-(tert-butyl)-6,7-dimethoxy-4H-3,1-benzoxazin-4-one, neoprzewaquinone A, and hesperetin 5-O-glucoside (Figure 7C). For the 208 differentially accumulated metabolites detected under the negative mode, the accumulation of 116 metabolites was upregulated, represented by sucralose, 6-Shogaol, 8-iso Prostaglandin A2, OxPE, OxPC, and 18-β-Glycyrrhetinic acid, while 92 metabolites were downregulated, mainly containing 8-iso Prostaglandin F2α Ethanolamide, xanthine, LPC 16:1, carminic acid, 4-((5-(4-Nitrophenyl)oxazol-2-yl)amino)benzonitrile, and N-Acetyl-D-alloisoleucine (Figure 7D). 

#### 3.2.3. Differential Metabolites between “Pink Variety” and “Violet Variety”

In total, 542 differentially accumulated metabolites existed between the “violet variety” and “pink variety” (Table S8). Among the 357 differentially accumulated “pos metabolites”, the accumulation of 188 and 169 metabolites was upregulated and downregulated, respectively. In particular, the most enriched “pos metabolites” contained betulonic acid, 4-Hydroxyalprazolam, salvianolic acid D, LPE 18:1, and carbamazepine-d10; the typical “neg metabolites” contained α-Cyclodextrin, Obacunone, Hydroxysafflor yellow A, and 2,6-Di-tert-butyl-1,4-benzoquinone (Figure 7E). In regard to the 185 differentially accumulated metabolites, 84 and 101 were upregulated and downregulated, respectively. In particular, 18-β-Glycyrrhetinic acid, 6-Shogaol, 4-(benzyloxy)benzoic acid, polygalaxanthone III, tulipanin, and 11-keto Testosterone (CRM) were the main “pos metabolites”; tenuifoliside B, maltopentaose, 6″-o-acetylgenistin, N-Acetyl-D-alloisoleucine, ornithine, and DL-Arginine were mainly enriched “neg metabolites” (Figure 7F). 

### 3.3. Integrative Analysis of Metabolome and RNA-seq Data

#### 3.3.1. The Enriched Pathways between Different Pairwise Comparisons

In total, 35 pathways were obtained through integrative analysis of metabolome data (“pos metabolites”) and RNA-seq data, while 63 pathways were enriched through integrative analysis of metabolome data (“neg metabolites”) and RNA-seq data (Table S9). In relation to the “metabolic pathways” (map01100), 237 differently expressed mRNA were involved, which led to the different accumulation of 39 metabolites (17 pos and 22 neg). Moreover, 179 DEGs participating in “biosynthesis of secondary metabolites” (map01110) were identified, which resulted in 26 differentially accumulated metabolites (11 pos and 15 neg). 

For the comparison of the “red variety” and “violet variety”, a list of 54 and 80 KEGG pathways were obtained through integrative analysis of RNA-seq data with “pos metabolites” and “neg metabolites”, respectively (Table S9). In regard to the representative “metabolic pathways” (map01100), “biosynthesis of secondary metabolites” (map01110), “microbial metabolism in diverse environments” (map01120), and “phenylpropanoid biosynthesis” (map00940), a total of 506, 317, 67, and 57 DEGs took part, leading to forty-one (twenty-three pos and eighteen neg), twenty-one (nine pos and twelve neg), thirteen (four pos and nine neg), and two (one pos and one neg) metabolites, respectively.

In regard to the comparison between the “pink variety” and “violet variety”, 56 and 43 KEGG pathways were enriched through comprehensive analysis of differentially expressed mRNAs with “pos metabolites” and “neg metabolites”, respectively (Table S9). A list of 447, 305, 59, and 53 DEGs participated in the “metabolic pathways” (map01100), “biosynthesis of secondary metabolites” (map01110), “phenylpropanoid biosynthesis” (map00940), and “microbial metabolism in diverse environments” (map01120), accounting for thirty-three, eleven, three, and nine differentially accumulated metabolites. 

#### 3.3.2. Integrative Analysis of Flavonoid Biosynthesis Pathway

**Figure 8:**
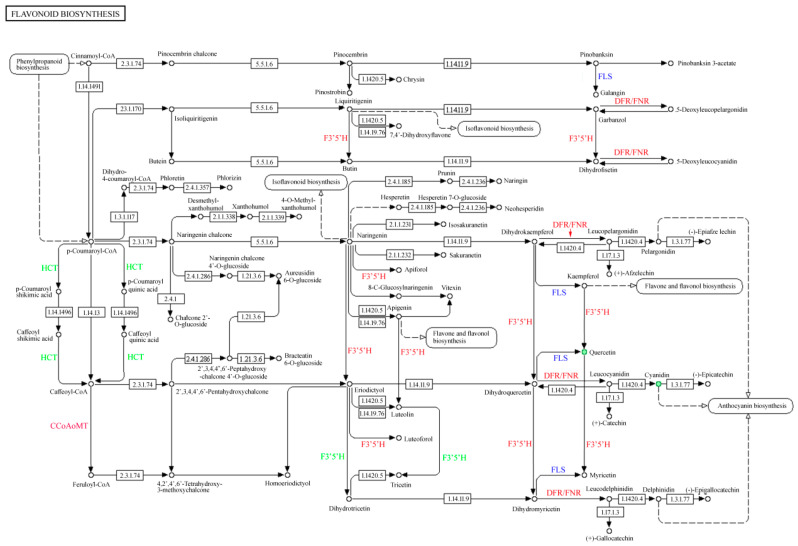
Overview of flavonoid biosynthesis process in *R. simsii* flowers. Notes: Numbers refer to corresponding enzymes listed in Enzyme Commission hierarchy. The red, blue, and green codes represent upregulated, downregulated, and unstable differentially expressed genes, respectively.

Compared with the “red variety”, 18 genes were differentially expressed, leading to four differentially accumulated metabolites, including quercetin, cyanidin, eriodictyol, and myricetin in the “pink variety”. In particular, the differential expression of shikimate *O-hydroxycinnamoyltransferase* (*HCT*) gene affected the accumulation of Caffeoyl-CoA; the upregulated expression of *caffeoyl-CoA O-methyltransferase* (*CCoAOMT*) gene might exert effects on Feruoyl-CoA synthesis in the “flavonoid biosynthesis” pathway (Figure 8). During the conversion of liquiritigenin into butin, the expression of *flavonoid 3′-monooxygenase* was upregulated, which might accelerate the catalytic process. In relation to the conversion of pinobanksin to galangin, dihydrokaempferol to kaempferol, and dihydromyricetin to myricetin, the expression of *flavonol synthase* (FLS) was all downregulated. Moreover, the upregulated expression of the *flavonoid 3′-monooxygenase* gene affected the branch synthesis pathway of garbanzol to dihydrofisetin. The upregulated expression of bifunctional dihydroflavonol 4-reductase/flavanone 4-reductase (DFR/FNR) accelerated five branch synthesis pathways, including the conversion of garbanzol and dihydrofisetin into 5-deoxyleucocyanidin, the conversion of dihydrokaempferol to leucopelargonidin, the conversion of naringenin to apiforol, the conversion of eriodictyol to luteoforol, and the conversion of dihydromyricetin to leucodelphinidin. With the comprehensive influence of F3′5′H and *flavonoid 3′-monooxygenase* genes, several metabolic pathways were significantly regulated, including the conversion of naringenin to eriodictyol, the conversion of apigenin to luteolin, the conversion of dihydrokaempferol to dihydroquercetin, and the conversion of kaempferol to quercetin. In addition, the upregulated expression of the *F3′5′H* gene affected four branch synthesis pathways: the conversion of eriodictyol to dihydrotricetin, the conversion of luteolin to tricetin, the conversion of dihydroquercetin to dihydromyricetin, and the conversion of quercetin to myricetin (Figure 8). 

Compared with the “red variety”, 29 DEGs were involved in “flavonoid biosynthesis” (map00941), leading to a different expression of quercetin and myricetin. In particular, the expression patterns of HCT, CCoAoMT, F3′5′H, FLS, and bifunctional DFR/FNR were almost the same in the *R. simsii* “violet variety”. However, it is worth mentioning that the expression levels of *CHS*, *leucoanthocyanidin reductase* (*LAR*), and *5-O-(4-coumaroyl)-D-quinate 3′-monooxygenase* genes varied largely between the “red variety” and “violet variety” (Figure 8). The upregulated expression of *CHS* accelerated the conversion of cinnamoyl-CoA to pinocembrin chalcone and p-Coumaroyl-CoA, the conversion of p-Coumaroyl-CoA to isoliquiritigenin and naringe chalcone, the conversion of dihydro-4-coumaroyl-CoA to phloretin, the conversion of caffeoyl-CoA to 2′,3,4,4′,6′-pentahydroxychalcone, and the conversion of feruloyl-CoA to 4,2′,4′,6′-tetrahydroxy-3-methoxychalcone. Furthermore, the downregulated expression of the *5-O-(4-coumaroyl)-D-quinate 3′-monooxygenase* gene affected the conversion of p-coumaroyl shikimic acid to caffeoyl shikimic acid and p-coumaroyl quinic acid to caffeoyl quinic acid, respectively. Additionally, the expression of the *LAR* gene could significantly affect the conversion of leucocyanidin to (+)-catechin (Figure 8). 

#### 3.3.3. Integrative Analysis of Flavone and Flavonol Biosynthesis Pathway

**Figure 9:**
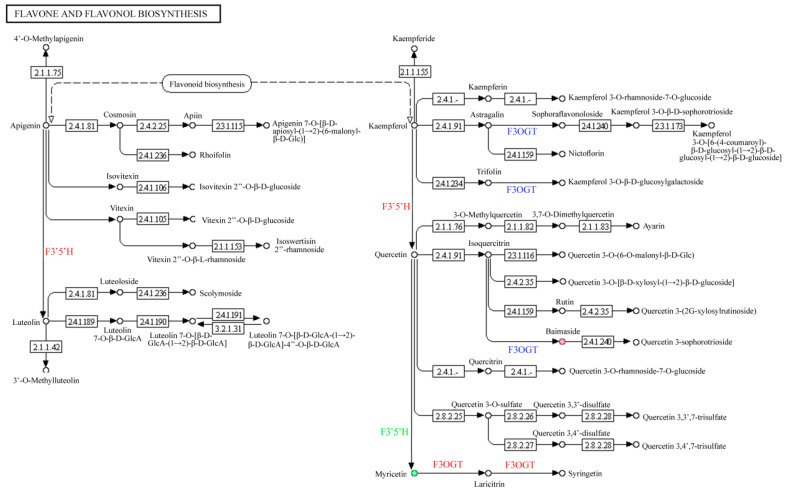
Overview of flavone and flavonol biosynthesis process in *R. simsii* flowers. Notes: Numbers refer to corresponding enzymes listed in Enzyme Commission hierarchy. The red, blue, and green codes represent upregulated, downregulated, and unstable differentially expressed genes, respectively.

Upon comparing the “red variety” and “pink variety”, six differentially expressed genes accounted for the different accumulation of quercetin, isovitexin, and laricitrin. The differentially expressed *F3′5′H* and *flavonoid 3′-monooxygenase* genes contributed a lot to the variation in flower color, involving the conversion of apigenin to luteolin and the conversion of kaempferol to quercetin in the “flavone and flavonol biosynthesis” pathway (Figure 9). Moreover, the differentially expressed F3′5′H also affected the conversion of quercetin to myricetin. The downregulated expression of flavonol-3-O-glucoside/galactoside glucosyltransferase (F3OGT) exerted great effects on three branch biosynthesis pathways, including the conversion of astragalin to sophoraflavonoloside, the conversion of trifolin to kaempferol 3-O-β-D-glucosylgalactoside, and the conversion of isoquercitrin to baimaside. However, expression of *F3OGT* gene was upregulated in the synthesis of syringetin from myricetin. 

Upon comparing the “red variety” and “violet variety”, nine DEGs were involved in “flavone and flavonol biosynthesis” (map00944), leading to the differentially accumulated laricitrin. The downregulated expression of F3′5′H and upregulated expression of *flavonoid 3′-monooxygenase* genes both contributed to the different accumulation of 3′-O-methylluteolin, quercetin, and myricetin (Figure 9). The regulated expression of F3OGT affected the conversion of astragalin to sophoraflavonoloside, the conversion of trifolin to kaempferol 3-O-β-D-glucosylgalactoside, and the conversion of isoquercitrin to baimaside. Moreover, the downregulated expression of the *F3OGT* gene first reduced the conversion of myricetin to laricitrin and then the conversion of laricitrin to syringetin. 

## 4. Discussion

As the largest genus of the family Ericaceae, *Rhododendron* contains 1025 species worldwide [25,26]. Due to various floral colors, like red, pink, purple, violet, blue, yellow and white, *Rhododendron* species are ideal materials to investigate the adaptive evolution of floral color traits [21]. For ornamental *Rhododendron* species, flower color is an important trait for breeding programs, which is controlled by pigment composition, pH, cell shape, and co-pigmentation [27,28]. As an adaptive trait, floral coloration is also commonly correlated with pollinators and natural environments [29]. Systematic identification of pigment composition and clarification of molecular mechanisms are vital, which are the foundation of genetic improvement. Up to now, research on *Rhododendron* floral coloration has mostly concentrated on evergreen azalea, mainly belonging to the subgenus *Tsutsusi*. Limited information on deciduous *Rhododendron* species could be obtained. 

Based on LC-MS/MS, the metabolites screened out from flowers of the natural deciduous shrub *R. simsii* contained alkaloids, benzenoids, lignans, neolignans and related compounds, lipids, lipid-like molecules, nucleosides, nucleotides, analogues, organic acids, organic nitrogen compounds, organic oxygen compounds, organoheterocyclic compounds, phenylpropanoids, and polyketides, inferring that flower colors were determined by the combined effects of secondary metabolites. In particular, 79 flavonoids (seven anthocyanidins, 42 flavanones, 10 flavans, 13 flavones, and seven flavonols) were successfully identified, which were less than those identified in *R. pulchrum* (149) with pink, white, and violet flowers [23]. Likewise, five anthocyanins and 23 flavonols were identified in mixed petals of *Rhododendron virgatum*, *Rhododendron nivale*, *Rhododendron aganniphum* var. Schizopeplum, *Rhododendron oreotrephes*, *Rhododendron faucium*, *Rhododendron vellereum*, *Rhododendron nyingchiense*, *Rhododendron hirtipes*, *Rhododendron wardii*, and *Rhododendron triflorum* [5]. Similarly, seven anthocyanins and 23 flavonols have been identified from thirty *Rhododendron* species, including subgenus *Hymenanthes* (sixteen species), subgenus *Tsutsusi* (six species), subgenus *Rhododendron* (three species), subgenus *Azaleastrum* (two species), subgenus *Pentanthera* (one species), subgenus *Rhodorastrum* (one species), and subgenus *Pseudorhodorastrum* (one species) [21].

Though six types of anthocyanins derivatives are common in vascular plants, only Cy and Dp were identified in the three wild *R. simsii* varieties with red, pink, and violet flowers. In particular, anthocyanin derivatives Dp, Pt, and Mv might contribute a lot to the violet and dark colors, while the derivatives Cy and Pg accounted for the bright-red color [22]. Similarly, Cy was the main anthocyanin found in flowers of *Rhododendron schlippenbachii* Maxim [20]. However, Pg, Cy, Dp, Pn, Pt, and Mv, were all detected in *R. pulchrum* flowers [23]. A high accumulation of anthocyanins was also identified in red flowers of *R. schlippenbachii* Maxim [20]. In Belgian hybrids of *R. simsii*, the most common anthocyanins are Cy 3-monoglucoside, Cy 3,5-diglucoside, and peonidin 3,5-diglucoside, while Mv 3,5-diglucoside and azaleatin 3-rhamnosylglucoside existed mainly in violet-colored *R. simsii* species and varieties [4]. Moreover, Mv 3-O-arabinoside-5-O-glucoside and Dp 3-O-arabinoside-5-O-glucoside are important for violet coloration [20]. The glycosylated derivatives of Mv and Dp were not detected. Differences in anthocyanin compositions might be due to species differences and differences in detection methods.

The variation existing in *R. simsii* flowers might be the comprehensive effect of multiple metabolic pathways. In particular, “flavone and flavonol biosynthesis”, “phenylalanine metabolism”, “tryptophan metabolism”, “ubiquinone and other terpenoid-quinone biosynthesis”, and “diterpenoid biosynthesis” might contribute a lot to flower color variation between the “red variety” and “pink variety”. The “biosynthesis of amino acids”, “aminoacyl-tRNA biosynthesis”, “2-oxocarboxylic acid metabolism”, “tropane, piperidine and pyridine alkaloid biosynthesis”, “cyanoamino acid metabolism”, and “pantothenate and CoA biosynthesis” might play important roles in color variation between the “violet variety” and “pink variety”. Furthermore, “tryptophan metabolism”, “arachidonic acid metabolism”, “phenylpropanoid biosynthesis”, “pantothenate and CoA biosynthesis”, “one carbon pool by folate”, and “phenylalanine metabolism” might account for the color variation between the “violet variety” and “pink variety”. Likewise, this phenomenon was present in *R. pulchrum*, *Sesamum indicum*, *Coreopsis tinctoria*, and Chinese jujube [30,31,32].

Genes, including *HCT*, *CCoAOMT*, *flavonoid 3′-monooxygenase*, *FLS*, *DFR/FNR*, *F3′5′H*, *CHS*, *LAR*, *O-(4-coumaroyl)-D-quinate 3′-monooxygenase* were differentially expressed among the three varieties, which ultimately led to the variation in flavonoid synthesis. The FLS protein, located in the nucleus and cytomembrane, could convert dihydrokaempferol and dihydroquercetin into kaempferol and quercetin, respectively [33]. In regard to the “flavone and flavonol biosynthesis pathway”, the expression of the genes *F3′5′H*, *flavonoid 3′-monooxygenase*, and *F3OGT* differed largely between different varieties, causing different accumulation of flavone and flavonols. In tobacco, pink flower color could be converted to dark purple through the heterologous expression of a pansy *F3′5′H* gene [34]. The CHS enzyme family, members of plant-specific type III polyketide synthase, participates in the synthesis of multiple secondary metabolites in plants, especially anthocyanin [35,36]. In particular, the *CHI*, *DFR*, *F3′5′H*, *F3H*, and *bHLH1* genes were all expressed in the flower and peel of *Solanum melongena* [37]. In the leaf of sweet potato, the silencing *FLS* gene converts the pink to purple through upregulating the expression of genes involved in the downstream pathway of anthocyanin biosynthesis, such as *DFR*, *ANS*, and *UFGT* genes, which increased the total anthocyanin content and reduced the total flavonol content [38,39].

Therefore, anthocyanin production in flowers of *R. simsii* could be changed through modifying genes involved in the branch of the phenylpropanoid pathway. This study will facilitate deep insights into the molecular regulatory mechanism governing flower color and provide an important basis for further genetic improvement of *R. simsii* varieties with novelty colors.

## Figures and Tables

**Table 1:** Anthocyanins and proanthocyanidins identified in petals of *R. simsii* varieties.

Compound Name	Formula	Molecular Weight	RT [min]	*m*/*z*	Ionization Modes	HMDB_ID	Lipidmaps_ID	SUB_CLASS	Description
Cyanidin	C15 H10 O6	286.04719	5.174	287.0545	Positive	HMDB0002708	LMPK12010002	Anthocyanidins	7-hydroxyflavonoids
Cyanidin 3-O-glucoside	C21 H21 Cl O11	484.07698	5.78	483.0697	Negative	-	LMPK12010110	Anthocyanidins	Anthocyanidin-3-O-glucoside
Procyanidin A2	C30 H24 O12	576.12578	5.334	577.13306	Positive	HMDB0037655	-	Proanthocyanidins	Biflavonoids and polyflavonoids
Procyanidin B1	C30 H26 O12	578.14219	5.01	577.13531	Negative	HMDB0029754	LMPK12030001	Proanthocyanidins	Biflavonoids and polyflavonoids
Procyanidin B2	C30 H26 O12	578.14398	5.022	579.15118	Positive	HMDB0033973	LMPK12030002	Proanthocyanidins	Biflavonoids and polyflavonoids
Tulipanin	C27 H31 O16	611.16144	4.989	610.15216	Negative	-	LMPK12010282	Proanthocyanidins	Anthocyanidins
Kuromanin	C21 H20 O11	448.09982	5.122	449.10693	Positive	HMDB0030684	-	Anthocyanidins	Anthocyanidin-3-O-glycosides
Delphinidin 3-sophoroside-5-rhamnoside	C33 H41 Cl O21	808.18242	5.734	809.19012	Positive	-	-	Anthocyanidins	Anthocyanidin-rhamnoside

## Data Availability

RNA-seq data of *R. simsii* varieties were deposited in the NCBI database with BioProject, Biosample, and SRA numbers of PRJNA998472, SAMN36704916-SAMN36704933, and SRR25433158- SRR25433172, respectively. All materials were conserved at the Huanggang Normal University Herbarium (Huanggang, Hubei Province, China).

## Supplementary Materials

The following supporting information can be downloaded at https://www.mdpi.com/article/10.3390/genes15081041/s1, Figure S1: The PCA analysis of metabolome data; Table S1: Information of metabolites identified in three *R. simsii* varieties; Table S2: HMDB annotation of metabolites; Table S3: LIPIDMAPS annotation of metabolites; Table S4: Flavonoids and derivatives identified in three *R. simsii* varieties; Table S5: Information of differentially accumulated metabolites in three *R. simsii* varieties; Table S6: Different metalites between ‘red variety ‘and ‘pink variety’; Table S7: Different metalites between ‘red variety’ and ‘violet variety’; Table S8: Different metalites between ‘pink variety’ and ‘violet variety’; Table S9: The enriched pathways between different *R. simsii* varieties through integrative analysis.

## Abbreviations

ANR	anthocyanidin reductase
ANS	anthocyanidin synthase
CCoAOMT	caffeoyl-CoA O-methyltransferase
CHI	chalcone isomerase
CHS	chalcone synthase
Cy	cyanidin
DFR/FN	dihydroflavonol 4-reductase/flavanone 4-reductase
DFR	dihydroflavonol 4-reductase
Dp	delphinidin
F3H	flavanone 3-hydroxylase
F3′H	flavonoid 3′-hydroxylase
F3′5′H	flavonoid 3′,5′-hydroxylase
FLS	flavonol synthase
F3OGT	flavonol-3-O-glucoside/galactoside glucosyltransferase
FPKM	fragments per kb per million mapped reads
HCT	shikimate O-hydroxycinnamoyltransferase
KEGG	Kyoto Encyclopedia of Genes and Genomes
LAR	leucoanthocyanidin reductase
Mv	malvidin
PAs	proanthocyanins
PAL	phenylalanine ammonia-lyase
PCA	principal component analysis
Pg	pelargonidin
Pn	peonidin
Pt	petunidin
QC	quality control
