# Supplementary figures and images for: Integrated Analyses of Metabolome and RNA-seq Data Revealing Flower Color Variation in Ornamental Rhododendron simsii Planchon
Source: Genes (Basel). 2024 Aug 7;15(8):1041. doi: 10.3390/genes15081041 (PMC11353987; doi:10.3390/genes15081041)

# PCA

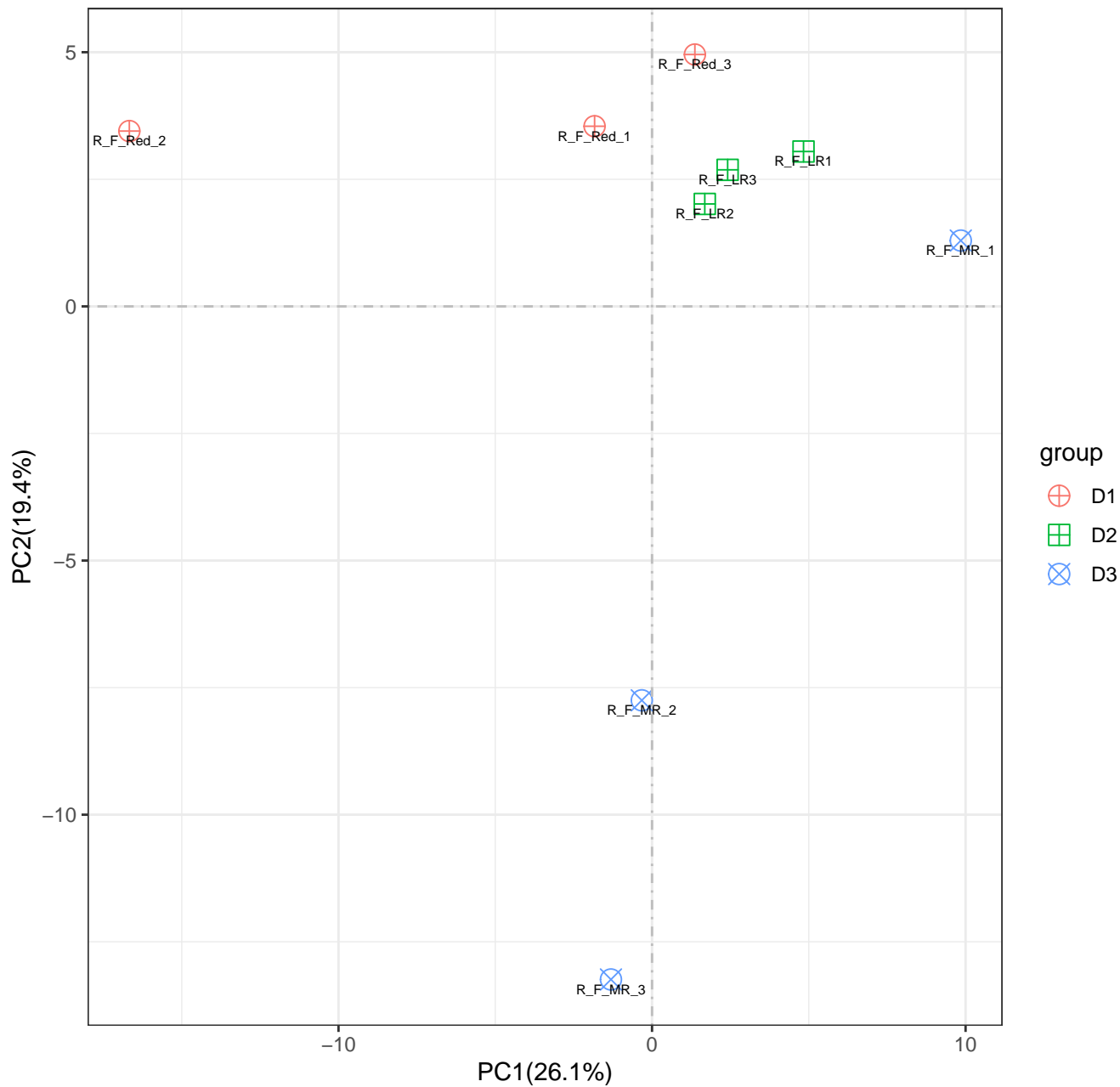

Supplement: Supplementary file 1 [file genes-15-01041-s001.zip › Figure S1.pdf]
